# Malignant pleural effusions and the role of talc poudrage and talc slurry: a systematic review and meta-analysis

**DOI:** 10.12688/f1000research.5538.2

**Published:** 2015-02-17

**Authors:** Srinivas Mummadi, Anusha Kumbam, Peter Y. Hahn

**Affiliations:** 1 Division of Pulmonary and Critical Care Medicine, Tuality Healthcare, Hillsboro, OR, 97123, USA; 2 Division of Pulmonary and Critical Care Medicine, Oregon Health & Science University, Portland, OR, 97239, USA; 3 Department of Internal Medicine, John H. Stroger, Jr. Hospital of Cook County, Chicago, IL, 60614, USA

**Keywords:** malignant pleural effusion, palliation, pleurodesis

## Abstract

**Background:** Malignant Pleural Effusion (MPE) is common with advanced malignancy. Palliative care with minimal adverse events is the cornerstone of management. Although talc pleurodesis plays an important role in treatment, the best modality of talc application remains controversial.

**Objective:** To compare rates of successful pleurodesis, rates of respiratory and non-respiratory complications between thoracoscopic talc insufflation/poudrage (TTI) and talc slurry (TS).

**Data sources and study selection:** MEDLINE (PubMed, OVID),  EBM Reviews (Cochrane database of Systematic Reviews, ACP Journal Club, DARE, Cochrane Central Register of Controlled Trials, Cochrane Methodology Register, Health Technology Assessment and NHS Economic Evaluation Database), EMBASE and Scopus. Randomized controlled trials published between 01/01/1980 - 10/1/2014 and comparing the two strategies were selected.

**Results: **Twenty-eight potential studies were identified of which 24 studies were further excluded, leaving four studies. No statistically significant difference in the probability of successful pleurodesis was observed between TS and TTI groups (RR 1.06; 95 % CI 0.99-1.14; Q statistic, 4.84). There was a higher risk of post procedural respiratory complications in the TTI group compared to the TS group (RR 1.91, 95% CI= 1.24-2.93, Q statistic 3.15). No statistically significant difference in the incidence of non-respiratory complications between the TTI group and the TS group was observed (RR 0.88, 95% CI= 0.72-1.07, Q statistic 4.61).

**Conclusions:** There is no difference in success rates of pleurodesis based on patient centered outcomes between talc poudrage and talc slurry treatments.  Respiratory complications are more common with talc poudrage via thoracoscopy.

## Introduction

Malignant Pleural Effusion (MPE) is a well described event in the natural history of advanced malignancy. Malignant etiology accounts for 22% of the diagnosed pleural effusions ^1^. Using data from the 2012 Nationwide Inpatient Sample (NIS), Healthcare Cost and Utilization Project (HCUP) and Agency for Healthcare Research and Quality, it is estimated that the aggregate charges (the “national bill”) were 722 million dollars in the USA ^2^.

Palliation with minimal adverse events remains the cornerstone of management ^3^. Talc pleurodesis and Indwelling Pleural Catheters (IPC) are the two most commonly used palliative approaches.

Talc pleurodesis can be achieved either by thoracoscopic instillation i.e.; talc insufflation/poudrage (TTI) or via a bedside chest tube i.e. talc slurry (TS). Existing systematic reviews concluded that thoracoscopic talc insufflation/poudrage was more efficacious when compared to bedside chest tube talc slurry ^4, 5^. New prospectively designed studies comparing TTI and TS have been published since then ^6– 8^. However the best initial approach for talc pleurodesis remains still unclear. To address the need for an update, a systematic review and meta-analysis of studies comparing thoracoscopic talc insufflation/poudrage and talc slurry in terms of patient centered outcomes was performed.

## Materials and methods

### Data sources and search

We conducted a systematic review with meta-analysis of studies undertaken between 01/01/1980 and 12/31/2014 using MEDLINE (PubMed, OVID), EBM Reviews (Cochrane database of Systematic Reviews, ACP Journal Club, DARE, Cochrane Central Register of Controlled Trials, Cochrane Methodology Register, Health Technology Assessment and NHS Economic Evaluation Database), EMBASE and Scopus. Unpublished data sets such as conference abstracts and ClinicalTrials.gov were also included in the full review phase to reduce the effect of publication bias ^9^.

The following keywords were used: *chemical pleurodesis, pleurodesis, talc pleurodesis, bedside pleurodesis, surgical pleurodesis, medical pleurodesis, thoracoscopicpleurodesis, thoracoscopic talc pleurodesis, thoracoscopicpoudrage, thoracoscopic talc poudrage, talc insufflation, thoracoscopic talc insufflation, pleuroscopy, medical thoracoscopy, talc poudrage, talc slurry, tube thoracostomy, chest tube talc slurry and malignant pleural effusion*. Both keywords and medical subject headings were used in a Boolean search strategy. An example search strategy can be found in the Appendix 1.

In addition, a pearl growing strategy was employed using frequently cited reviews of malignant pleural effusion treatments. They were included to be analyzed in the full review phase of the study. Approval from the Institutional Review Board was unnecessary because this is a meta-analysis.

### Study selection

Inclusion and exclusion criteria were framed prior to the implementation of the search strategy. To evaluate outcomes in adult malignant pleural effusion patients (18 + years) undergoing talc pleurodesis, we included studies based on the following criteria:

1) A randomized design was used in studying talc pleurodesis in patients with malignant pleural effusion between 01/01/1980 and 12/31/2014.
2) Patients undergoing bedside TS were compared with patients undergoing thoracoscopic talc insufflation/poudrage (TTI) in the above fashion.
3) Sufficient outcomes data were reported [Efficacy of pleurodesis, respiratory complications and non-respiratory complications].

Non-English publications, case reports and series, pediatric studies, descriptive studies without a control group, retrospective studies and prospective controlled studies without randomization were excluded. Eligible articles were reviewed by two reviewers for inclusion; disagreements were resolved via discussion. An examination of the full-length articles was carried with the intent of eliminating duplicate studies or same patient cohorts.

### Data extraction and outcome measures

Two reviewers independently extracted and rated the data from the selected full length articles using a standardized form. From each study, the data abstracted included study name/year, study design (prospective controlled, randomized controlled trial, retrospective etc.), cancer cell type, patient inclusion criteria, sample sizes for the bedside/surgical pleurodesis arms, technique employed in the bedside/surgical arms and the follow-up schedule.

Outcomes data pertaining to pleurodesis efficacy, respiratory complications, and non-respiratory complications were also extracted.

Talc pleurodesis for recurrent malignant pleural effusion is a palliative procedure and does not aim to have a mortality benefit. Therefore, measuring mortality outcomes was not the focus of the meta-analysis.

Various endpoints (pleurodesis failure vs success; radiological recurrence vs further need for pleural procedures) have been used to measure efficacy of the intervention in prior studies ^6, 10^.

To define efficacy, we chose to measure the success rates of pleurodesis rather than failure rates due to the relative ease of applicability of this measure in the real world clinical setting. A successful pleurodesis was defined *a priori* as accompanied by the lack of a need for repeat pleural procedures. Where clearly defined, asymptomatic radiological recurrences were included in the “successful” group (three asymptomatic recurrences in a total of four recurrences in the study by Yim *et al.*, 1996).

Respiratory complications were defined as occurrence of respiratory conditions such as pneumonia, Acute Respiratory Distress Syndrome (ARDS), acute respiratory failure, re-expansion pulmonary edema, bronchospasm, empyema, pulmonary embolism, prolonged air leak, bronchopleural fistula, atelectasis requiring bronchoscopy and subcutaneous emphysema.

Immediate non-respiratory complications were tabulated from the complications mentioned in the full length articles. These included fever, wound infection, chest pain, tumor recurrence at site, myocardial infarction, need for blood transfusions, arrhythmias and immediate post procedural death.

### Quality assessment criteria

The randomized controlled trials that met inclusion criteria were evaluated for quality using components of the modified Jadad scale ^11^. The presence of the following features was appraised:

A) A description of the study confirming the randomized nature.
B) Method of allocation to the study arms described and whether adequate/inadequate.
C) Description of withdrawals and dropouts.

Due to the nature of the comparison (surgical vs bedside procedure), we felt that other features of the scale (description of a double blind nature) could not be appraised during our quality assessment. Two raters independently determined the quality of the studies included. Disagreements were resolved by discussions and final consensus.

### Statistical analysis

Outcomes data for successful pleurodesis, respiratory and non-respiratory complications were summarized using descriptive statistics (simple count, proportion of the study sample). They were visually presented in Forest plots. The Mantel-Haenszel method ^12^ was used to combine data from individual studies and the results were reported as pooled relative risks (RR). Heterogeneity among the studies included was investigated by performing the I ^2^ test ^13^. Meta-analyses were conducted using the fixed effects model when heterogeneity between studies was low (I ^2^ < 40%) and the random effects model otherwise ^9^.

To confirm the robust nature of the results, a sensitivity analysis was performed by removing one study at a time and determining the outcome.

Publication bias was examined by visually examining the filled funnel plots using trim and fill method. Other methods (Begg’s correlation ^14^ and Egger’s linear regression intercept) ^15^ were additionally used.

All analyses were performed using a statistical software package (Comprehensive Meta-Analysis, version 2.2.064; Biostat, Englewood, NJ).

## Results

**Figure 1.**
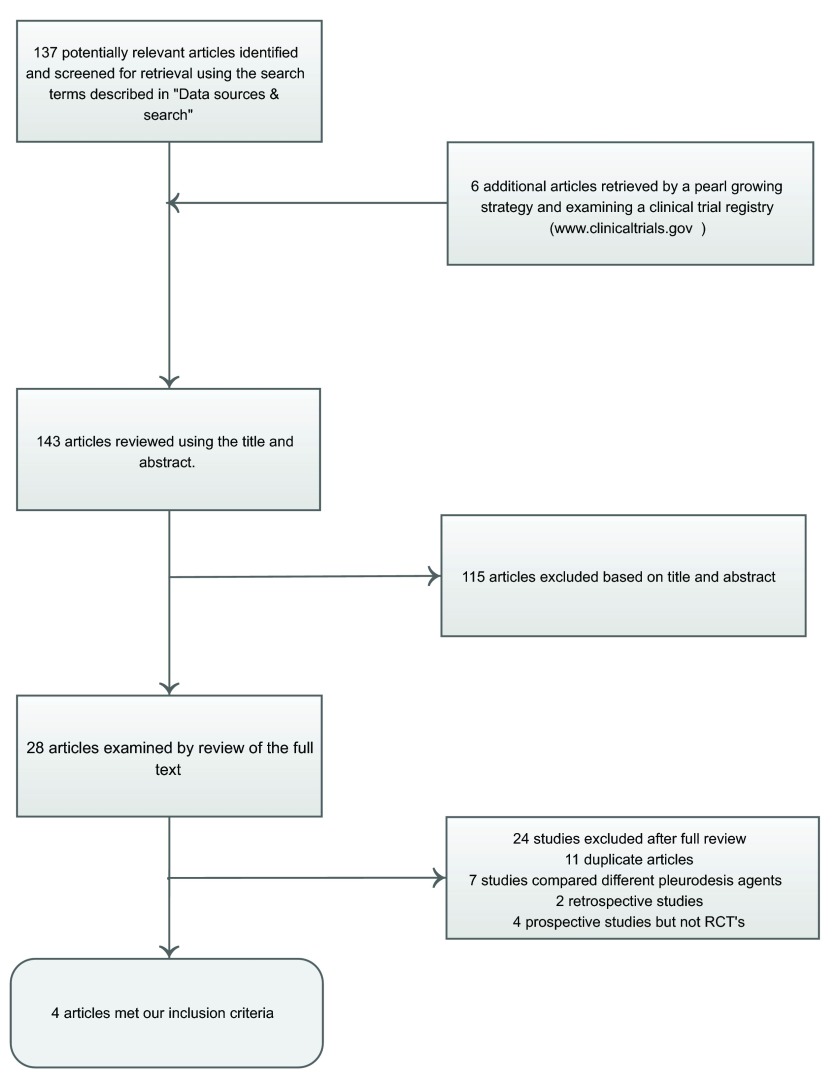
Flowsheet of study selection process.

Based on initial search, 137 articles were obtained and reviewed independently by two reviewers. Pearl growing strategy was employed to seek additional articles and resulted in five articles. A clinical trial registry ( www.ClinicalTrials.gov) was also examined and resulted in one additional article. These 143 articles were reviewed and 115 articles were excluded based on title and abstract. A total of 28 potential studies were thus identified with our search strategy. Twenty-four studies were further excluded, leaving four studies ^6, 10, 16, 17^ for the final analysis. The sequence describing the above process can be found in Figure 1.

None of the studies restricted the study population to a single cancerous cell type.

None of the included studies employed thoracoscopic evacuation of the malignant pleural effusion prior to bedside TS insertion via a chest tube.

Follow-up periods varied through the studies (Range = 30–425 days). Where available, recurrence data for the most distal available time point were selected for the meta-analysis.

All of the studies included in the analysis underwent quality assessment. The average Jadad score ^11^ was 1.5 out of a maximum possible score of 4 (Ranges 1–2). Out of the possible seven ways to assess the quality ^11^, only four questions could be answered due to the nature of the intervention. It was not possible reliably or ethically for the original investigators to have carried out efficient blinding in a surgical versus bedside clinical experiment.

## Measures of successful pleurodesis

**Figure 2.**
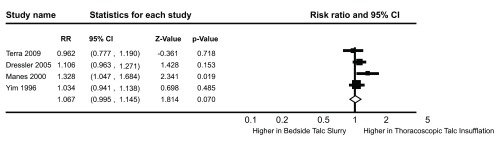
Pooled relative risks (RRs) of success rates post talc pleurodesis. *RR*, *risk ratio*, *CI*, confidence interval.

**Figure 3.**
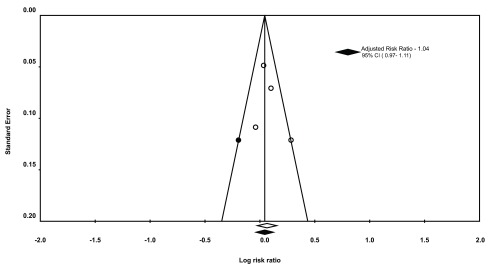
Filled funnel plot using the trim and fill method for succesful pleurodesis rates post talc pleurodesis: imputed studies - ●, observed studies - ○, *CI* – confidence interval.

The results of the pooled RR are shown in Figure 2. The four studies included in this analysis enrolled a total of 454 patients with malignant pleural effusion ( Table 1). There was no statistically significant difference in the proportion of successful pleurodesis between the bedside TS (successful pleurodesis/patients who underwent pleurodesis, n/N = 167/218 pts) and the TTI groups (successful pleurodesis/patients who underwent pleurodesis, n/N = 197/236 pts, pooled RR 1.06; 95% CI 0.99-1.14; Q statistic, 4.84; I ^2^ statistic, 38%). There was no evidence of publication bias (P-value = 0.49 for the Begg’s test, P-value= 0.54 for the Egger’s regression intercept). After using the trim and fill methodology ( Figure 3), these results did not change (RR- 1.04, 95% CI = 0.97-1.11, Q statistic, 9).

**Table 1.** Characteristics of studies comparing rates of successful pleurodesis

Study/Year Country	Intervention Design	Cancer Type	Definition of Success	Successful Pleurodesis in TTI Group n/N, (%)	Successful Pleurodesis in TS group n/N, (%)	Follow up schedule	Quality score	Quality problems
Terra/2009 Brazil	TTI vs TS RCT	All cancers	Lack of both symptoms and further need for pleural procedures	25/30 (83.3%)	26/30 (86.6%)	1,3,6 months followed by q3 months or if symptoms arose	2	Allocation process unclear
Dresler/2005 USA	TTI vs TS RCT	All cancers	No radiological recurrence	119/152 (78.2%)	92/130 (70.7%)	1-6 months	2	Allocation process unclear
Manes*/2000 Spain	TTI vs TS RCT	All cancers	Not defined but recurrences randomized to further pleural procedures	25/26 (96.1%)	21/29 (72.4%)	1-12 months	1	Inappropriate allocation process, Potential “recycling” of patients into intervention arms
Yim/1996 China	TTI vs TS RCT	All cancers	No radiological recurrence, however symptomatic patients who needed further procedures clearly identified	28/28 (100%)	28/29 (96.5%)	q6 weeks from 1-4.5 months, then q3 months	2	Allocation process unclear

*Study published only as an abstract form TTI, Thoracoscopic talc insufflation, Also known as Thoracoscopic talc poudrage TS, Talc slurry applied via a bedside chest tube RCT, Randomized Controlled Trials q- Every

The definitions of an efficacious pleurodesis intervention varied in the included studies. Three studies ^10, 16, 17^ reported procedure failure by measuring clinically significant recurrences and one study reported the number of successful procedures defined as the lack of recurrence. This study defined recurrence based on radiological data alone ^6^. The remainder of the studies clearly mentioned the number of patients who were symptomatic and required further pleural procedures once a recurrent pleural effusion was diagnosed ^10, 16, 17^.

A sensitivity analysis pooling data from studies reporting only clinically significant recurrences was performed leaving out one study ^6^. This did not result in a different statistical outcome (pooled RR 1.07; 95% CI 0.92-1.25; Q statistic, 4.49; I ^2^ statistic 55.53%).

As follow-up periods varied widely, a sensitivity analysis pooling the data from studies reporting 30 day outcomes was carried out and did not result in a different statistical outcome.

### Risk of respiratory complications

**Figure 4.**
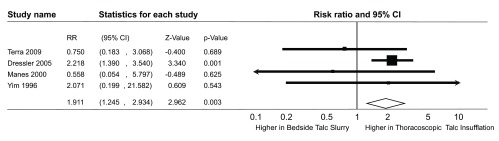
Pooled relative risks (RRs) for respiratory complications post talc pleurodesis. *RR*, *risk ratio*, *CI*, confidence interval.

The results of the pooled RR are shown in Figure 4. The four studies included in this analysis reported outcomes on a total of 591 patients who underwent talc pleurodesis for palliation of malignant pleural effusion ( Table 2).

**Table 2.** Characteristics of studies included for studying risk of respiratory complications

Study/Year Country	Intervention Design	Talc description	Anesthesia	Respiratory complications	Incidence in TTI Group n/N, (%)	Incidence in TS Group n/N, (%)	Quality score	Quality problems
Terra/2009 Brazil	TTI vs TS RCT	Noncalibrated talc (Mean diameter = 25 μm, 10% of the particles had a diameter < 10 μm)	TTI- General anesthesia TS- IV, Local anesthesia	Pneumonia, Pulmonary edema, Subcutaneous emphysema	3/30 (10%)	4/30 (13.3%)	2	Allocation process unclear
Dresler/2005 USA	TTI vs TS RCT	Non calibrated talc	TTI- General anesthesia TS- N/A	Empyema, BP fistula, Atelectasis, Pneumonia, Respiratory failure, PE	53/223 (23.7%)	21/196 (10.7%)	2	Allocation process unclear
Manes*/2000 Spain	TTI vs TS RCT	N/A	TTI- Local anesthesia TS- Local anesthesia	Empyema, Bronchospasm	1/29 (3.4%)	2/29 (6.8%)	1	Inappropriate allocation process, Potential “recycling” of patients into intervention arms
								
Yim/1996 China	TTI vs TS RCT	Purified talc from the U.K, no information on calibration	TTI-General anesthesia TS-Local anesthesia	Acute respiratory failure, Reexpansion pulmonary edema, Persistent air leak	2/28(7.1%)	1/29 (3.4%)	2	Allocation process unclear

*Study published only as an abstract form TTI, Thoracoscopic talc insufflation, also known as Thoracoscopic talc poudrage TS, Talc slurry via a bedside chest tube RCT, Randomized Controlled Trials N/A, Not available U.K, United Kingdom IV, Intravenous BP fistula, Bronchopleural fistula PE, Pulmonary Embolism

**Figure 5.**
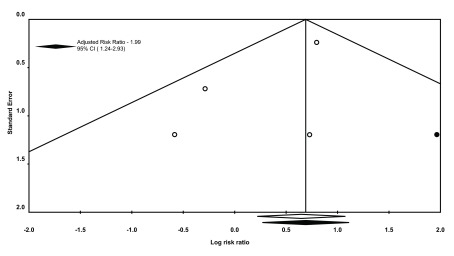
Filled funnel plot using the trim and fill method for risk of respiratory complications: imputed studies - ●, observed studies - ○, *CI* – confidence interval.

There was a statistically significant higher risk of post procedural respiratory complications in the TTI group (Incidence of respiratory complications/Pts who underwent pleurodesis, n/N = 59/307 pts) compared to the TS group (Incidence of respiratory complications/Pts who underwent pleurodesis, n/N = 28/284 pts, pooled RR 1.91, 95% CI= 1.24-2.93, Q statistic 3.15, I ^2^ statistic 4.79%). There was no evidence of publication bias (P-value = 1.0 for the Begg’s test, P-value= 0.24 for the Egger’s regression intercept). After using the trim and fill methodology ( Figure 5), these results did not change (RR- 1.99, 95% CI = 1.30-3.04, Q statistic, 4.32).

A sensitivity analysis pooling data from studies with ≥ 2 score on the Modified Jadad scale was performed leaving out one study with a score of 1 ^17^. This did not result in a different statistical outcome (pooled RR 1.99, 95% CI= 1.29-3.08, Q statistic 2.05, I ^2^ statistic 2.49).

### Risk of non-respiratory complications

**Figure 6.**
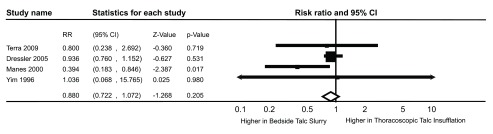
Pooled relative risks (RRs) for non-respiratory complications post talc pleurodesis. *RR*, *risk ratio*, *CI*, confidence interval.

The results of the pooled RR are shown in Figure 6. The four studies included in this analysis reported outcomes on a total of 591 patients who underwent talc pleurodesis for palliation of malignant pleural effusion ( Table 3).

**Table 3.** Characteristics of studies included for studying risk of non-respiratory complications

Study/Year Country	Intervention Design	Immediate non respiratory complications	Incidence in TTI Group n/N, (%)	Incidence in TS Group n/N, (%)	Quality score	Quality problems
Terra/2009 Brazil	TTI vs TS RCT	Fever, Wound infection, prolonged drainage	4/30 (13.3%)	5/30 (16.6%)	2	Allocation process unclear
Dresler/2005 USA	TTI vs TS RCT	Fever, Wound infection, RBC transfusion, Dysrhythmia, MI, DVT, Immediate post procedural death	99/223 (44.3%)	93/196 (47.4%)	2	Allocation process unclear
Manes*/2000 Spain	TTI vs TS RCT	Fever, Chest pain	6/26 (23%)	17/29 (58.6%)	1	Inappropriate allocation process, Potential “recycling” of patients into intervention arms
Yim/1996 China	TTI vs TS RCT	Tumor recurrence at wound site, Wound infection	1/28 (3.5%)	1/29 (3.4%)	2	Allocation process unclear

*Study published only as an abstract form TTI,Thoracoscopic talc insufflation, Also known as Thoracoscopic talc poudrage TS, Talc slurry applied via a bedside chest tube RCT, Randomized Controlled Trials RBC- Red Blood Cell MI-Myocardial Infarction DVT- Deep Venous Thrombosis

**Figure 7.**
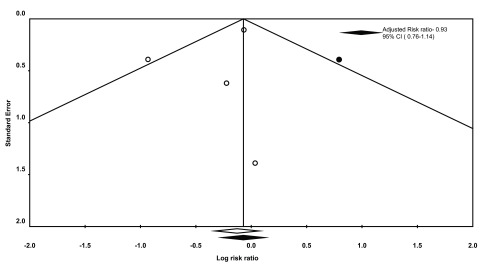
Filled funnel plot using the trim and fill method for risk of non-respiratory complications: imputed studies - ●, observed studies - ○, *CI* – confidence interval.

There was no statistically significant difference in the incidence of non-respiratory complications between the TTI group (Incidence of non-respiratory complications/Pts who underwent pleurodesis, n/N = 110/307 pts) and the TS group (Incidence of non-respiratory complications/Pts who underwent pleurodesis, n/N = 116/284 pts, pooled RR 0.88, 95% CI = 0.72-1.07, Q statistic 4.61, I ^2^ statistic 34.96%). There was no evidence of publication bias (P-value = 1.0 for the Begg’s test, P-value = 0.48 for the Egger’s regression intercept). After using the trim and fill methodology ( Figure 7), these results did not change (RR- 0.93, 95% CI = 0.76-1.12, Q statistic, 9.8).

A sensitivity analysis pooling data from studies with ≥2 score on the Modified Jadad scale was performed leaving out one study with a score of 1 ^17^. This did not result in a different statistical outcome (pooled RR 0.93, 95% CI= 0.76-1.14, Q statistic 0.06, I ^2^ statistic 0.0).

## Discussion

Many experts believe that serial thoracentesis is not an ideal choice for treating the recurrent malignant pleural effusion ^18, 19^.

Talc pleurodesis was first performed in 1935 ^20^ and is still commonly employed in the treatment of malignant pleural effusions. Although studies have shown talc to be the best chemical agent in terms of pleurodesis success and risk of recurrence ^21, 22^, the best method of applying talc remains controversial. Our meta-analysis demonstrates that both talc poudrage (TTI) and talc slurry (TS) offer similar rates of efficacy. There was no difference in the rates of successful pleurodesis (i.e., lack of need for further pleural procedures or symptoms). TTI did have a greater risk of respiratory complications. There was, however, no difference in the rate of non-respiratory complications such as fever and need for blood transfusions.

Our results are in contrast to those of previous meta-analyses ^5^, including the recently withdrawn Cochrane analysis which suggested improved success rates of talc pleurodesis utilizing TTI. The conclusion of these analyses was that thoracoscopic pleurodesis with talc was the optimal method for pleurodesis in patients with malignant pleural effusions. However, several newer prospective studies have been published since ^6, 7^ and have been incorporated into the present analysis.

Arguments in favor of TTI include the observation that there is more complete lung expansion after the procedure ^18^. This is certainly understandable given that take-down of adhesions is typically performed during the procedure itself as opposed to TS. Interestingly, Terra *et al.* using CT scanning post-TTI and TS to assess degree of post procedure lung expansion did not find a correlation between clinical outcomes and initial degree of lung expansion ^7^. These authors postulated that factors other than the degree of visceral and parietal pleura apposition were important in determining the success of pleurodesis. Likewise, there are no data to substantiate an existing notion that TTI would result in a superior dispersion of talc in the pleural space. Mager *et al.* used 99m Tc-labeled talc to show that rotation protocols did not affect the overall dispersion of talc suspensions after TS ^23^. The degree of dispersion also did not affect pleurodesis success ^23^.

In comparing TTI and TS, several difficulties arise. Pleurodesis success rates vary in the literature, due to the inconsistent definition of pleurodesis success and failure used in different studies. Failure or recurrence has been defined radiologically in some studies ^6^ but it has been argued that patient centered outcomes such as new symptoms and need for further pleural procedures are more pertinent outcomes ^7^. In our meta-analysis, we determined success *a priori* as the lack of need for further pleural procedures and disregarded asymptomatic radiological recurrences where possible.

The technique of both TS and TTI vary significantly between centers and this is evident in the included studies. TS varied in regard to length of chest tube clamping, rotating or not-rotating the patient, size of chest tube, and timing of chest tube removal. With regard to TTI, in three of the four studies TTI was performed under general anesthesia and the ability to tolerate general anesthesia was in fact an entry criteria. Overall, 88% of patients in our analysis underwent general anesthesia for TTI. One could argue that the increased respiratory complications observed with TTI may be related to general anesthesia and single lung ventilation. Despite the concerns of ARDS with the use of ungraded talc, the studies included in our meta-analysis did not report specific cases of ARDS. Non-specific respiratory failure was, however, reported in patients in the study by Dresler ^6^ and Yim *et al.* reported a case of acute respiratory failure in the TS group ^16^.

With the increasing numbers of interventional pulmonologists performing pleuroscopy (medical thoracoscopy) ^24^ under local and/or moderate sedation, the question of which procedure is the most optimal for talc pleurodesis is increasingly relevant. Whether talc poudrage performed during pleuroscopy with local or moderate sedation and dual lung ventilation is equivalent to surgical thoracoscopy (VATS) in terms of pleurodesis success and complications is unknown. Further studies are needed to compare talc poudrage performed with pleuroscopy versus TS.

One may wonder whether the question of TTI versus TS is still relevant in the era of indwelling pleural catheters (IPCs). Certainly, in the patient with trapped lung, both TS and TTI would likely be ineffective and indeed all of the studies in this meta-analysis excluded patients with possible trapped lung physiology. In the patient with malignant effusion without trapped lung, however, clear superiority of IPCs has not been demonstrated ^3^. In fact, talc pleurodesis may be more economical compared to IPC in patients with good performance status and projected life expectancy of >6 weeks ^25, 26^. The issue of cost is especially relevant in the era of health care reform and accountable care organizations. With the advent of newer molecular/hormonal therapies especially in breast cancer, malignant pleural effusion is increasingly recognized as a non-terminal event ^27^. Perhaps most importantly, patient preference is paramount ^28^ and no study has clearly demonstrated the superiority of IPCs compared to talc pleurodesis.

Our study has several limitations. The results of meta-analyses are dependent on the quality of the studies included. Of note, one of the included studies (Manes *et al.*, 2000) has several potential quality concerns as reflected by a modified Jadad score of 1. These include existing only in an abstract form, inappropriate randomization allocation and possible recycling of patients into treatment arms. We included this study due to its unique conclusion (TTI is superior to TS in terms of efficacy) and a large treatment effect. Reassuringly, sensitivity analysis performed by leaving this study out did not change the final estimates of all the studied outcomes. The inclusion of only randomized controlled trials was necessary due to significant bias inherent in non-randomized prospective studies. Despite the lack of heterogeneity between studies, the individual studies varied substantially (technique of talc pleurodesis, varying definitions of recurrence and follow-up schedule). Sensitivity analyses performed leaving out one study at a time did not impact the results, suggesting robust data. Publication bias is an inherent limitation of meta-analyses. It is reassuring to see that accounting for it did not result in a statistically significant departure from the original point estimates.

In conclusion, our meta-analysis demonstrates that there is no difference in success rates of pleurodesis based on patient centered outcomes between talc poudrage and talc slurry. Respiratory complications are more common with talc poudrage via thoracoscopy. Further studies are needed, however, to look at the role of talc pleurodesis via pleuroscopy. The decision of which procedure to perform needs to take into account also the patient preferences.

## Figures and Tables

**Table:** PRISMA 2009 Checklist

Section/topic	#	Checklist item	Reported on Page
**TITLE**			
Title	1	Identify the report as a systematic review, meta-analysis, or both.	**Page 1**
**ABSTRACT**			
Structured summary	2	Provide a structured summary including, as applicable: background; objectives; data sources; study eligibility criteria, participants, and interventions; study appraisal and synthesis methods; results; limitations; conclusions and implications of key findings; systematic review registration number.	**Page 1**
**INTRODUCTION**			
Rationale	3	Describe the rationale for the review in the context of what is already known.	**Page 3**
Objectives	4	Provide an explicit statement of questions being addressed with reference to participants, interventions, comparisons, outcomes, and study design (PICOS).	**Page 3**
**METHODS**			
Protocol and registration	5	Indicate if a review protocol exists, if and where it can be accessed (e.g., Web address), and, if available, provide registration information including registration number.	**N/A**
Eligibility criteria	6	Specify study characteristics (e.g., PICOS, length of follow-up) and report characteristics (e.g., years considered, language, publication status) used as criteria for eligibility, giving rationale.	**Page 3**
Information sources	7	Describe all information sources (e.g., databases with dates of coverage, contact with study authors to identify additional studies) in the search and date last searched.	**Page 3**
Search	8	Present full electronic search strategy for at least one database, including any limits used, such that it could be repeated.	**Appendix 2**
Study selection	9	State the process for selecting studies (i.e., screening, eligibility, included in systematic review, and, if applicable, included in the meta-analysis).	**Page 3**
Data collection process	10	Describe method of data extraction from reports (e.g., piloted forms, independently, in duplicate) and any processes for obtaining and confirming data from investigators.	**Page 3**
Data items	11	List and define all variables for which data were sought (e.g., PICOS, funding sources) and any assumptions and simplifications made.	**Page 3**
Risk of bias in individual studies	12	Describe methods used for assessing risk of bias of individual studies (including specification of whether this was done at the study or outcome level), and how this information is to be used in any data synthesis.	**Pages 3–4**
Summary measures	13	State the principal summary measures (e.g., risk ratio, difference in means).	**Pages 4–7**
Synthesis of results	14	Describe the methods of handling data and combining results of studies, if done, including measures of consistency (e.g., I2) for each meta-analysis.	**Pages 4–7**
Risk of bias across studies	15	Specify any assessment of risk of bias that may affect the cumulative evidence (e.g., publication bias, selective reporting within studies).	**Pages 4–5**
Additional analyses	16	Describe methods of additional analyses (e.g., sensitivity or subgroup analyses, meta- regression), if done, indicating which were pre-specified.	**Pages 4–7**
**RESULTS**			
Study selection	17	Give numbers of studies screened, assessed for eligibility, and included in the review, with reasons for exclusions at each stage, ideally with a flow diagram.	**Figure 1, Pages 4–5**
Study characteristics	18	For each study, present characteristics for which data were extracted (e.g., study size, PICOS, follow-up period) and provide the citations.	**Tables 1–3**
Risk of bias within studies	19	Present data on risk of bias of each study and, if available, any outcome level assessment (see item 12).	**Pages 4–7**
Results of individual studies	20	For all outcomes considered (benefits or harms), present, for each study: (a) simple summary data for each intervention group (b) effect estimates and confidence intervals, ideally with a forest plot.	**Figures 2,4,6**
Synthesis of results	21	Present results of each meta-analysis done, including confidence intervals and measures of consistency.	**Pages 4–7**
Risk of bias across studies	22	Present results of any assessment of risk of bias across studies (see Item 15).	**Pages 4–7**
Additional analysis	23	Give results of additional analyses, if done (e.g., sensitivity or subgroup analyses, meta- regression [see Item 16]).	**Pages 4–7**
**DISCUSSION**			
Summary of evidence	24	Summarize the main findings including the strength of evidence for each main outcome; consider their relevance to key groups (e.g., healthcare providers, users, and policy makers).	**Pages 7,9,10**
Limitations	25	Discuss limitations at study and outcome level (e.g., risk of bias), and at review-level (e.g., incomplete retrieval of identified research, reporting bias).	**Page 10**
Conclusions	26	Provide a general interpretation of the results in the context of other evidence, and implications for future research.	**Page 11**
**FUNDING**			
Funding	27	Describe sources of funding for the systematic review and other support (e.g., supply of data); role of funders for the systematic review.	**Page 11**

*From:* Moher D, Liberati A, Tetzlaff J, Altman DG, The PRISMA Group (2009). Preferred Reporting Items for Systematic Reviews and Meta-Analyses: The PRISMA Statement. PLoS Med 6(6): e1000097. doi:10.1371/journal.pmed1000097

## Appendix 1

### Search strategy for MEDLINE using PubMed

1. Search “talc poudrage” [MeSH OR tw] – 162 results
2. Search “chemical pleurodesis” [MeSH OR tw] – 266 results
3. Search “pleurodesis” [MeSH OR tw] – 2154 results
4. Search “thoracoscopic pleurodesis” [MeSH OR tw] – 52
5. Search “thoracoscopic talc pleurodesis” [MeSH OR tw] -42
6. Search “thoracoscopic poudrage” [all terms] – 87
7. Search “thoracoscopic talc poudrage” [MeSH OR tw] – 49
8. Search “talc insufflation” [MeSH OR tw] – 57
9. Search “thoracoscopic talc insufflation” [MeSH OR tw] – 19
10. Search “pleuroscopy” and “talc pleurodesis” [All Fields] – 329
11. Search “pleuroscopy” and “talc insufflation” [All Fields] – 69
12. Search “pleuroscopy” and “talc poudrage” [All Fields] – 119
13. Search “talc slurry” [MeSH OR tw] – 95
14. Search 1 OR 2 OR 3 OR 4 OR 5 OR 6 OR 7 OR 8 OR 9 10 OR 11 OR 12 OR 13 – 2206 results
15. Search “malignant pleural effusion” [MeSH OR tw] -3426 results
16. Search “malignant pleuritis” [MeSH OR tw] -29
17. Search 14 and 15 – 580 results. Subsequently, the following filters were applied [Clinical trials including Phase I–IV, Controlled Clinical Trials, Randomized Controlled Trials, Reviews, Systematic Reviews, Meta-Analyses, observational studies, abstract availability, publication dates from 01/01/1989 to 12/31/2014, adult human studies, English-language publications] – 71 results.
18. Search 14 and 16. Filters applied [Clinical trials including Phase I–IV, Controlled Clinical Trials, Meta-Analyses, observational studies, randomized controlled trials, systematic reviews, review articles, abstract availability, publication dates from 01/01/1989 to 10/1/2014, adult human studies, English-language publications] – 0 results.
Terms such as thoracoscopic poudrage, chest tube talc slurry, thoracostomy talc slurry, chest tube talc pleurodesis, talc slurry sclerosis did not reveal any results in the [MeSH OR tw] category.
