# Supplementary material for: Malignant pleural effusions and the role of talc poudrage and talc slurry: a systematic review and meta-analysis
Source: F1000Res. 2015 Feb 17;3:254. Originally published 2014 Oct 27. [Version 2] doi: 10.12688/f1000research.5538.2 (PMC4382843; doi:10.12688/f1000research.5538.2)
Supplement: Peer review history

# Peer review: Referee response for version 2

**DOI:** 10.5256/f1000research.6583.r6534

**Published:** 2015-03-27

**Authors:** Fabien Maldonado

**Affiliations:** 1 Division of Pulmonary and Critical Care Medicine, Mayo Clinic, Rochester, MN, USA

This is a very well-written and conducted systematic review and metaanalysis.

I would have liked to get a sense of the level of agreement between the two reviewers for study inclusion, though I suspect the search was relatively straightforward.

It would be worthwhile pointing out in the discussion that the Dresler paper weighs heavily in the results, particularly with respect to respiratory complications, inherent in surgical procedures and perhaps, in some instances, unrelated to the talc pleurodesis itself. The methodological flaws of this study (exclusion of a substantial number of patients after randomization limiting the benefits of randomization) should have been specifically highlighted in my opinion.

Nonetheless, this is an important meta-analysis that should be indexed.

I have read this submission. I believe that I have an appropriate level of expertise to confirm that it is of an acceptable scientific standard.

# Peer review: Referee response for version 1

**DOI:** 10.5256/f1000research.5917.r7143

**Published:** 2014-12-31

**Authors:** Pyng Lee

**Affiliations:** 1 Division of Respiratory and Critical Care, National University Hospital, Singapore, Singapore

Malignant pleural effusion is also one of the leading causes of exudative effusion; studies have demonstrated that 42 to 77% of exudative effusions are secondary to malignancy ( Marel *et al.*,1993; Valdés *et al.*,1996)

Need statistician review.

I have read this submission. I believe that I have an appropriate level of expertise to confirm that it is of an acceptable scientific standard.

# Peer review: Referee response for version 1

**DOI:** 10.5256/f1000research.5917.r6535

**Published:** 2014-10-31

**Authors:** Gary Lee

**Affiliations:** 1 Pleural Medicine Unit, The Lung Institute of Western Australia, Nedlands, WA, Australia

**Overview:**

The topic is important and clinically relevant. Talc pleurodesis is commonly practiced. The debate on the superiority of thoracoscopic poudrage (TTI) versus talc slurry (TS) has been ongoing for decades. A meta-analysis incorporating the most recent data is useful.

The methodology appears sound. The four selected articles are well described and acknowledged in most review articles as the only randomized studies directly addressing this topic.

The interpretation of the data is fair and balanced. The endpoints chosen are clinically relevant and important.  Most other reviews or book chapters on the issue have only focused on the success/failure rate of the pleurodesis.  The authors rightly highlighted the increased complication rates of TTI.  The figures and graphs are clear and easy to understand.

The discussion is based on the analyses and the postulations for the observed results are sound and reasonable.  

Overall this is an excellent paper and I would support its indexing.

There are several few points that the authors may wish to consider. I believe that this article may be improved if the authors can address the following:

1. **The study by Manes *et al.* (2000)**
  The authors quite rightly included not only peer reviewed papers but also all published abstracts. This approach is according to standard meta-analysis practice, in order to avoid publication bias such that ‘negative’ studies (often not published in full) are not excluded. However, including data from published abstracts preclude scrutiny of the detailed methods, analyses and thus raises questions on the quality of the results. The situation in the meta-analysis of TTI vs TS highlights the pros vs cons of including data that were not peer reviewed.
  Three of the four selected studies were published in respected journals and were subjected to peer-reviewing processes. The study by Manes however was published only as an abstract 14 years ago, and to date had not been published as a full paper. This by itself raises great concerns. The results of this particular study deviated significantly from all the other three. The authors have identified clear methodological concerns (especially ‘recycling’ of patients into randomization after failing pleurodesis). Unless the authors have obtained details from the primary research group, it is doubtful that a short abstract could provide adequate details for proper critique of the methods and results. Including this study without qualifying its many limitations may distort the interpretation of the readers.
  I suggest that the authors should:
  ​
  1. Highlight the point that the Manes study was never published in full in the text/legends;
  2. Perform and show a separate analysis excluding the Manes study;
  3. Discuss the rationale of including/excluding this single study in the Discussion section.
    I believe the above measures are justified as the Manes study was not a ‘negative’ study and would never have been biased against if ever submitted for full publication.
    ​
2. **An alternate way of presenting the data**
  Although it would not change the actual conclusion or the raw data, presenting the results as ‘success’ rather than ‘failure’ rates would quite significantly change the ‘visual effects’ of the graphs. Take for example the Dresler study. The RR for failure is 0.74 but if expressed as ratio of success rates it would become 1.11, the Yim study 1.04 and the Terres study 0.96. This probably presents a more useful interpretation for clinicians and patients – that the magnitude of superiority of TTI in any of the studies is at best 1.11 times over TS (excluding Manes *et al.*). 
3. **Discussion**
  It would be useful to include that there are no scientific grounds why insufflation should be more advantageous than slurry. Talc does not work as a glue (otherwise we would have had major problems when talc was still included in baby nappy powders).  Even distribution of talc over the pleural surface is not therefore critical. Radio-active isotope studies have shown that talc, even when applied as a slurry, can distribute around the pleural cavity via respiratory motions.

I have read this submission. I believe that I have an appropriate level of expertise to confirm that it is of an acceptable scientific standard, however I have significant reservations, as outlined above.
